# Three months of melatonin treatment reduces insulin sensitivity in patients with type 2 diabetes—A randomized placebo‐controlled crossover trial

**DOI:** 10.1111/jpi.12809

**Published:** 2022-06-09

**Authors:** Esben S. Lauritzen, Ulla Kampmann, Mette G. B. Pedersen, Lise‐Lotte Christensen, Niels Jessen, Niels Møller, Julie Støy

**Affiliations:** ^1^ Steno Diabetes Center Aarhus Aarhus University Hospital Aarhus Denmark; ^2^ Medical/Steno Research Laboratory, Department of Clinical Medicine Aarhus University Aarhus Denmark; ^3^ Department of Molecular Medicine Aarhus University Hospital Aarhus Denmark; ^4^ Department of Clinical Pharmacology Aarhus University Hospital Aarhus Denmark; ^5^ Department of Biomedicine Aarhus University Aarhus Denmark

**Keywords:** indirect calorimetry, insulin secretion, insulin sensitivity, insulin signaling and rs10830963, melatonin, sleep

## Abstract

The use of the sleep‐promoting hormone melatonin is rapidly increasing as an assumed safe sleep aid. During the last decade, accumulating observations suggest that melatonin affects glucose homeostasis, but the precise role remains to be defined. We investigated the metabolic effects of long‐term melatonin treatment in patients with type 2 diabetes including determinations of insulin sensitivity and glucose‐stimulated insulin secretion. We used a double‐blinded, randomized, placebo‐controlled, crossover design. Seventeen male participants with type 2 diabetes completed (1) 3 months of daily melatonin treatment (10 mg) 1 h before bedtime (M) and (2) 3 months of placebo treatment 1 h before bedtime (P). At the end of each treatment period, insulin secretion was assessed by an intravenous glucose tolerance test (0.3 g/kg) (IVGTT) and insulin sensitivity was assessed by a hyperinsulinemic‐euglycemic clamp (insulin infusion rate 1.5 mU/kg/min) (primary endpoints). Insulin sensitivity decreased after melatonin (3.6 [2.9–4.4] vs. 4.1 [3.2–5.2] mg/(kg × min), *p* = .016). During the IVGTT, the second‐phase insulin response was increased after melatonin (*p* = .03). In conclusion, melatonin treatment of male patients with type 2 diabetes for 3 months decreased insulin sensitivity by 12%. Clinical use of melatonin treatment in dosages of 10 mg should be reserved for conditions where the benefits will outweigh the potential negative impact on insulin sensitivity.

## 1 INTRODUCTION

Melatonin is a tryptophan‐derived indolamine secreted by the pineal gland as part of a circadian rhythm.^1^ The rhythm is entrained by retinal light input resulting in peak concentrations during nighttime.^1^ Physiologically, melatonin is involved in the regulation of sleep^2^ and body core temperature.^3^ During the last two decades, melatonin has also been proposed to be involved in the regulation of glucose homeostasis by mainly two lines of evidence. First, several genome‐wide associations studies (GWAS) have reported, that the frequent rs10830963 single‐nucleotide polymorphism, located in the melatonin receptor 2 gene (MTNR1B), which may be a gain‐of‐function variant, is strongly associated with elevated fasting glucose and risk of type 2 diabetes.^4^, ^5^, ^6^, ^7^, ^8^ Second, acute daytime administration of melatonin results in 10%–25% decreased insulin sensitivity.^9^, ^10^, ^11^ On the other hand, carriers of rare loss‐of‐function variants in the MTNR1B and individuals with low nightly melatonin secretion have increased risk of type 2 diabetes.^12^, ^13^ As the use of melatonin is increasing as a sleep aid in both adults^14^ and children,^15^ it is advantageous to clarify the metabolic effects of long‐term melatonin treatment on glucose metabolism. A number of placebo‐controlled randomized studies have addressed this question, but the outcomes have been limited to glycated hemoglobin (HbA1c) levels, fasting plasma glucose and insulin levels, mostly with beneficial effects.^16^ We recently performed a systematic review and meta‐analysis of the metabolic consequences of melatonin treatment in healthy individuals and patients with metabolic diseases and found no effects of treatment on fasting plasma glucose, but reduced fasting insulin levels and a tendency towards increased insulin sensitivity measured by homeostatic model assessment for insulin resistance (HOMA‐IR).^16^ However, none of the previous long‐term placebo‐controlled randomized studies stratified on rs10830963 genotype and none of the studies evaluated the metabolic effects of melatonin treatment beyond fasting glucose and insulin levels. Thus, a comprehensive assessment of the metabolic effects of melatonin treatment with direct measurements of insulin sensitivity and β‐cell function in patients with type 2 diabetes stratified on rs10830963 genotype is warranted.

## 2 METHODS

### 2.1 Trial design

The study was a double‐blinded, randomized, placebo‐controlled, crossover study.

### 2.2 Eligibility criteria for participants

#### 2.2.1 Inclusion criteria

Clinical diagnosis of type 2 diabetes within the last 20 years, male sex, Caucasian race, age 40–70 years, body mass index 25–35 kg/m^2^ at diagnosis.

#### 2.2.2 Exclusion criteria

More than three daily antihypertensive drugs, blood pressure > 160/100, insulin treatment, > 3 daily antidiabetic drugs, > 1 lipid‐lowering drug, HbA1c > 65 mmol/mol, serious chronic illness, shift work within the last year, travel across more than four time zones planned within the next 6 months, use of melatonin on a regular basis, diagnosis of a sleep disorder, alcohol or substance abuse, melatonin allergy, daily consumption of medicine that interacts with the pharmacokinetics of melatonin or medical treated depression or anxiety disorders within the last 3 years.

### 2.3 Settings

The ambulatory visits and the study days were conducted at Aarhus University Hospital in a thermoneutral environment.

### 2.4 Interventions

Participants underwent two 12‐week treatment periods separated by 4 weeks with either 10 mg melatonin (M) (Glostrup Pharmacy) or placebo tablets (P) 1 h before bedtime (Supporting Information: Figure S1). Each treatment period included two ambulatory visits and one study day at the end of each period. After inclusion, no participants changed their daily medication. Participants were instructed not to change dietary or exercise patterns throughout the entire study.

### 2.5 Ambulatory visits

During the first ambulatory visit in each treatment period, the study medicine was dispensed and routine biochemistry was controlled, a second ambulatory visit was held after 6 weeks of treatment to ensure continued adherence to the study drug, for reporting of adverse effects or changes in the general health of the participant.

### 2.6 Preparation for study days

The week before the study days, participants were instructed to maintain a regular wake‐sleep cycle with bedtimes between 10 and 12 pm and wake‐up times between 6 and 8 a.m. To assess sleep quality and quantity, participants wore an ActiGraph (wGT3X‐BT) on their nondominant wrist. Also, participants were instructed to avoid intake of alcohol and caffeine and strenuous physical exercise 48 h before the study days. Participants fasted from 10 pm before the study days.

### 2.7 Study days

**Figure 1:**
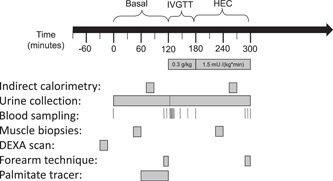
Outline of the study day. Outline of the study day. IVGTT, intravenous glucose tolerance test (0.3 g glucose/kg body weight), HEC, hyperinsulinemic‐euglycemic clamp insulin infusion rate 1.5 mU/(kg body weight × min), DEXA scan, dual‐energy X‐ray absorptiometry.

On the study days (Figure 1), participants arrived by car at 08:00 a.m. (*t* = −30 min). After arrival, body composition was evaluated with a whole‐body DEXA scan (Holologic, Discovery). At *t* = 0 min, the participants were instructed to empty their bladder and afterward three intravenous (iv) cannulas were inserted, one in a right dorsal hand vein, one in the right antecubital vein for infusions, and one in a retrograde fashion in the left antecubital vein for deep venous blood sampling. The right hand was subsequently placed under a heating blanket for arterialized blood sampling.^17^

### 2.8 Palmitate tracer

To evaluate systemic palmitate turnover, a 1‐h infusion of 9,10‐[^3^H]‐palmitate tracer (PerkinElmer/Department of Clinical Physiology and Nuclear Medicine, Aarhus University Hospital) was started (0.3 µCi/min) at *t* = 60 min. Systemic palmitate turnover was calculated as previously described.^18^

### 2.9 Intravenous glucose tolerance test (IVGTT) and hyperinsulinemic‐euglycemic clamp (HEC) procedures

At *t* = 120 min an IVGTT, for determination of β‐cell function, was started by infusing 0.3 g glucose/kg from a 20% glucose solution over 2 min. At *t* = 120–130 min blood samples were drawn with 2‐min intervals (first‐phase insulin secretion, FPIS) and again at *t* = 140, 160 and 180 min (second‐phase insulin secretion, SPIS). At *t* = 180 min an HEC procedure,^19^ for determination of insulin sensitivity, was commenced by infusion of 1.5 mU/(kg × min) insulin (Humulin Regular; Eli Lilly). Initially, the glucose levels declined to 5.0 mmol/l and were clamped at this level for the rest of the study day with a variable infusion of a 20% glucose solution containing 20 mmol/l of KCl. Combining an IVGTT with a subsequent HEC has been validated previously.^20^

### 2.10 Indirect calorimetry

At *t* = 70 min indirect calorimetry was performed for 15 min to assess the respiratory quotient (RQ) and energy expenditure (EE) (Oxycon Pro; CareFusion). The indirect calorimetry was repeated for 15 min at *t* = 250 min during the HEC. Urine was sampled from *t* = 0–120 min and from *t* = 120–300 min to determine urea excretion. Free fatty acid (FFA) and glucose oxidation rates were calculated by adjusting for protein oxidation.^21^

### 2.11 Forearm glucose uptake and FFA exchange

From *t* = 100–120 and *t* = 280–00 (HEC) min arterialized and venous blood samples were drawn with 10‐min intervals for calculation of forearm glucose uptake and FFA exchange with the use of concomitant occlusion strain‐gauge plethysmography.^22^

### 2.12 Blood samples

Plasma glucose was instantly measured using a YSI 2300 STAT plus (YSI Life Sciences). Serum insulin, C‐peptide and glucagon were measured by ELISA (Mercodia; RRID:AB_2877672, RRID:AB_2750847 and RRID:AB_2892203). FFA was analyzed with a commercial kit (Wako Chemicals). Plasma melatonin was analyzed by RIA (IBL; RRID:AB_2892203). All of the above were analyzed in batch. Routine biochemical analyses were analyzed at the Department of Biochemistry, Aarhus University Hospital.

### 2.13 Muscle biopsies

Muscle biopsies were obtained with a Bergström needle at *t* = 50 (basal) and at *t* = 230 (HEC) min in local anesthesia. Muscle biopsies were prepared and analyzed with western blot analysis as previously described.^23^ The primary antibodies used are described in the supplementary material.

### 2.14 Genotyping

The genotyping of the MTNR1B intronic SNP (NM_005959.3:c.223 + 5596 C > G [rs10830963]) was performed using Sanger Sequencing as previously described.^24^

### 2.15 Outcomes

The primary outcomes were changes in insulin sensitivity derived from the HEC, changes in insulin secretion (quantified as the FPIS and SPIS during the IVGTT), the effect of the rs10830963 genotype on these two outcomes and changes in insulin signaling in muscle biopsies. Secondary outcomes were changes in body composition, EE, substrate metabolism, palmitate turnover, forearm glucose, and palmitate uptake and changes in a broad panel of hormones.

### 2.16 Sample size and participants

The sample size was derived from power calculations using the glucose infusion rate during the HEC. With a power of 0.8, an *α* of .05, and an expected standard deviation of the difference of 12%, 18 participants were required to detect a difference of 8.5% between placebo and melatonin treatment. Detailed flowchart for the participants is available in Supporting Information: Figure S2. No participants changed their antidiabetic, antihypertensive, or lipid‐lowering therapy during the study period.

### 2.17 Randomization and blinding

The randomization was performed by Glostrup Pharmacy at www.randomization.com 1:1 in a bloc of 20 (due to dropouts, another 1:1 bloc randomization of six was performed). The allocation was concealed by delivery of sequentially numbered identical drug containers in pairs (period one and period two). The allocation sequence was provided in a sealed opaque envelope, which was broken after the last participant completed the last study day. Thus, both participants and the investigator were blinded during the entire trial.

### 2.18 Study periods

Recruitment was initiated in July 2019, the first participant started treatment in august 2019 and the last participant completed the last study day in April 2021.

### 2.19 Compliance

Compliance to treatment was evaluated with counting of remaining pills in the drug containers. The participants omitted their study medication 4 (0–9) % of the time while treated with melatonin and 1 (0–5) % of the time on placebo treatment (*p* = .12).

### 2.20 Questionnaires

Psychological well‐being was assessed with World Health Organization Five Well‐being Index (WHO‐5)^25^ and with Major Depression Inventory (MDI)^26^ at both ambulatory visits and study days. Daytime sleepiness was assessed with Epworth Sleepiness Scale (ESS),^27^ sleep quality was assessed with Pittsburgh Sleepiness Scale (PSQI),^28^ and morningness‐eveningness type was assessed with Morningness‐Eveningness Questionnaire (MEQ)^29^ during the study days.

### 2.21 Statistics

All statistical work was performed in STATA 16 (StataCorp). Normal distribution of data was assessed by inspection of quantile‐quantile (QQ) plots. Log‐transformation was performed when appropriate. Normally distributed data are presented as means [95% confidence intervals], not normally distributed data are presented as medians (25%–75% range). Comparisons between two means/medians were performed with paired two‐tailed *t*‐tests/Wilcoxon signed‐rank test. In case of repeated measurements, a mixed model was used with intervention (placebo or melatonin), time (different time points during the study day), order of treatment (melatonin‐placebo or placebo‐melatonin), visit (1st visit or 2nd visit), genotype (risk‐allele carrier or homozygous wild‐type carrier) and interaction between time, genotype and intervention as fixed effects and with study participants and the interaction between study participants and visit as random effects. In case of no interaction, the additive model was used. The validity of the mixed model was assessed by inspection of QQ‐plots of the standardized residuals and scatter plots of the standardized residuals versus the fitted values. Log‐transformation was performed when appropriate. If the assumptions underlying the validity of the linear mixed model were not fulfilled, incremental areas under the curve (iAUC) were calculated by cubic spline approximation.^30^ To obtain the order‐of‐treatment effect, the mixed model was also applied on the iAUC data. Both the linear mixed model and the iAUC calculations were pre‐specified in the protocol. The insulin sensitivity (M‐value) was calculated as the mean glucose infusion rates (mg/[kg × min]) during the last 30 min of the clamp. Missing values (forearm glucose and FFA uptake *n* = 4/34 during basal and 5/34 during clamp, muscle biopsies *n* = 5/34 during basal and 5/34 during clamp) were ignored in the mixed model. Graphical work was performed with SigmaPlot 11 (Systat Software). Order‐of‐treatment effects are reported for the primary outcomes (all *p* > .1). There was no significant order‐of‐treatment effect for the secondary outcomes (all *p* > .05).

### 2.22 Data and resource availability

The datasets generated during and/or analyzed during the current study are available from the corresponding author upon reasonable request.

## 3 RESULTS

### 3.1 Study participants

For baseline characteristics of the study participants please see Table 1.

**Table 1:** Baseline characteristics of the 17 participants

	All participants	G‐allele carriers (*n* = 9)	C‐allele carriers (*n* = 8)	*p *Value
Age (years)	65 (41–70)	65 (54–66)	62 (54–68)	.72
Height (m)	1.79 ± 0.07	1.79 ± 0.07	1.80 ± 0.08	.94
Weight (kg)	95 ± 14	91 (87–102)	96 (87–106)	.54
BMI (kg/m^2^)	29 ± 3.5	29 ± 3	29 ± 4	.93
Total cholesterol (mM)	3.8 (3.2–5.5)	4.1 (3.7–4.9)	3.6 (3.5–4.1)	.2
High‐density lipoprotein cholestrol (mM)	1.2 ± 0.3	1.2 ± 0.2	1.2 ± 0.3	.65
Low‐density lipoprotein cholestrol (mM)	2.1 ± 0.7	2.3 ± 9,7	2.0 ± 0.7	.39
Triglycerides (mM)	1.7 (0.6–3)	2.1 (0.9–2.4)	1.5 (0.8–1.8)	.23
HbA1c (mmol/mol)	48.5 ± 7.0	45.6 ± 5.8	51.9 ± 7.0	.06
*Antidiabetic drugs*				
Metformin	16 (94)	9 (100)	7 (88)	.28
SGLT2‐inhibitors	3 (18)	2 (22)	1 (13)	.63
DPP‐IV inhibitors	6 (35)	3 (33)	3 (38)	.83
GLP‐1 agonists	1 (6)	0 (0)	1 (13)	.26
*Anticoagulants, antiplatelet, and lipid‐lowering drugs*				
NOAC	2 (12)	1 (11)	1 (13)	.90
Acetylsalicylic acid 75 mg	1 (6)	1 (11)	0 (0)	.33
Lipid‐lowering drugs (statins)	9 (52)	2 (22)	7 (88)	.007
*Antihypertensive drugs*				
RAAS inhibitors	7 (41)	4 (44)	3 (38)	.80
Thiazides	1 (6)	0 (0)	1 (13)	.26
β‐blockers	2 (12)	2 (22)	0 (0)	.16
Calcium channel blockers	2 (12)	2 (22)	0 (0)	.16

*Note*: Baseline characteristics of the participants as either medians (25%–75%), means ± SD, or number receiving therapy (percentage of total participants) stratified on rs10830963 genotype.

Abbreviations: BMI, body mass index; DPP, dipeptidyl peptidase; GLP, glucagon‐like peptide; HbA1c, glycated hemoglobin; NOAC, novel oral anticoagulants; RAAS, renin‐angiotensin‐aldosterone system; SGLT2, sodium‐glucose cotransporter.

### 3.2 Genotypes

Eight participants were heterozygous carriers of the risk G allele, eight participants were homozygous carriers of the wildtype C allele and one participant was homozygous carrier of the risk G allele.

### 3.3 Insulin sensitivity

**Figure 2:**
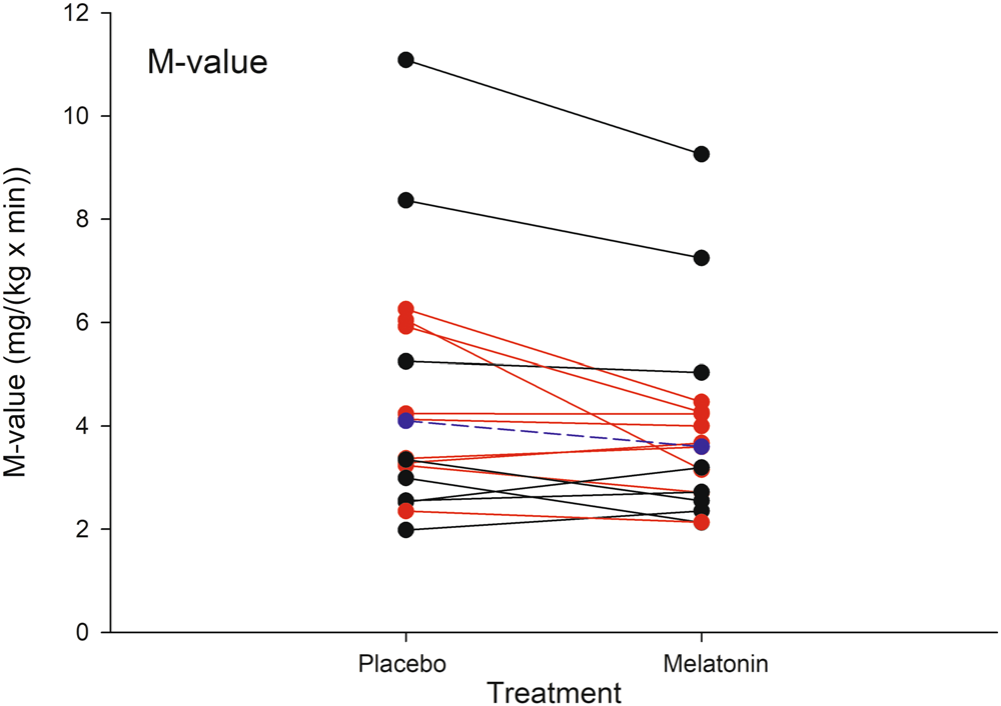
M‐value. M‐value after 3 months of melatonin treatment and 3 months of placebo treatment. Two black circles connected by the same black line represent a participant who is a homozygous carrier of the rs10830963 wild‐type allele. Two red circles connected by the same red line represent a participant who is an rs10830963 risk‐allele carrier. The blue circles connected by the blue dashed line represent the geometric means of all 17 participants. *p, 0.016.

The M‐value (Figure 2) decreased significantly after M compared with P (3.6 [2.9–4.4] vs. 4.1 [3.2–5.2], difference −0.6 [−1.13 to −0.13] mg/(kg × min), *p* = .016). There was no interaction between genotype and treatment (*p* = .54) and there was no main effect of genotype (*p* = .42). In addition, there were no order‐of‐treatment effect (*p* = .32). 

### 3.4 Body composition and bones

Total fat content, total lean body mass, body weight, total bone mineral content, total bone mineral density (BMD), lumbar spine BMD, and total hip BMD were not significantly different after M compared with P (Table 2).

**Table 2:** Body composition and bones

	Placebo	Melatonin	Difference	*p*
Total bone mineral content (kg)	2.784 [2645–2924]	2.825 [2.657–2993]	0.041 ± [−0.065 to0.146]	.43
Total BMD (g/cm3)	1.149 (1.11–1.25)	1.161 (1.12–1.124)	0.017 [−0.015 to 0.049]	.29
Lumbar spine BMD (g/cm3)	1.072 [1.010–1.134]	1.055 [0.974–1.14]	−0.017 [−0.077 to 0.0434]	.56
Total hip BMD (g/cm3)	1.248 [1.189–1.307]	1.271 [1.200–1.341]	0.021 [−0.023 to 0.068]	.31
Total fat content (g)	31654 [27 279–36 030]	32094 [28 310–35 878]	−49 (−551 to 1544)	.64
Total lean mass (g)	62571 [59 165–65 977]	63267 [59 990–66 543]	459 (−70 to 1368)	.17
Body weight (g)	94226 [87 333–101 121]	95361 [89 143–10 1578]	947 (−539 to 2080)	.13

*Note*: Means [95% CI] or medians (25%–75%) for body composition and bone health variables. *p* values are from paired *t*‐tests between melatonin and placebo.

### 3.5 Melatonin levels

Melatonin levels were measured at *t* = 0 and *t* = 120 min. Melatonin levels were significantly increased after M compared with P (treatment effect *p* < .001; melatonin *t* = 0 min: 52 [30–77], *t* = 120 min: 32 [17–45], placebo *t* = 0 min: 7 [4–10], *t* = 120 min: 4 [3–6] pg/ml). In addition, there was a significant interaction between genotype and treatment (*p* = .03). After placebo treatment, there was no significant difference in melatonin levels between risk and wildtype carriers (effect of genotype, *p* = .44), but after melatonin treatment, risk carriers had 3.8 (1.7–8.5) fold higher melatonin levels compared with wildtype carriers (*p* = .002). There was no correlation between the individual reduction in M‐value and individual difference in melatonin levels at *t* = 120 min between melatonin and placebo (*r* ^2^ = 0.007, *p* = .75).

### 3.6 Glucose, insulin, C‐peptide, and glucagon during IVGTT

**Figure 3:**
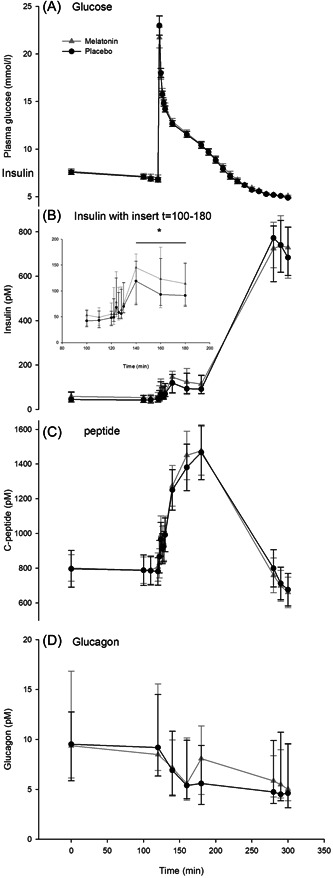
Glucose, insulin, C‐peptide, and glucagon during the study days. Gray triangles connected by gray lines are after 3 months of melatonin treatment and dark circles connected by dark lines are after 3 months of placebo treatment. Panel A: Glucose levels during the study day. Panel B: Insulin levels during the study day with time = 100‐180 inserted for better visualization of the insulin response during the IVGTT. Panel C: C‐peptide levels during the study day. Panel D: Glucagon levels during the study day. Panels A and C are means ± standard error, panel B and D are medians (25%–75% ranges).

The levels over time of glucose, insulin, C‐peptide, and glucagon are shown in Figure 3A–D. Normally distributed residuals were not achievable for insulin, glucagon, and C‐peptide despite log transformations. Therefore, these variables were only analyzed with iAUCs (Supporting Information: Table S1). There were no statistically significant differences in iAUC for glucose, C‐peptide, or glucagon between M and P but insulin iAUC from time 130 to 180 min was significantly increased after melatonin treatment compared to placebo (*p* = .03) and C‐peptide had the same tendency (*p* = .1) (Supporting Information: Table S1). For glucose, there were no statistically significant interactions between genotype, time, and treatment and no statistically significant main effects of genotype or treatment. In addition, there was no order‐of‐treatment effect (120–130 min *p* = .91 and 130–180 min *p* = .9). For C‐peptide, glucagon, and insulin, there were no effects of the genotype between or within groups (data not shown). In addition, there was no order‐of‐treatment effects for C‐peptide (120–130 min *p* = .12 and 130–180 min *p* = .64) and insulin (120–130 min *p* = .12 and 130–180 min *p* = .10).

### 3.7 Forearm glucose uptake and FFA exchange

FFA exchange and glucose uptake over the forearm were not affected by M: Glucose (basal: M −0.062 [−0.465 to 0.342] P 0.291 [0.019–0.563] µmol/[100 ml forearm × min]; clamp: M 2.54 [1.02–4.06] P 2.65 [1.39–3.91] µmol/[100 ml forearm × min], time (basal or clamp) × treatment *p* = .54, time *p* = .000, and treatment *p* = .99). FFA (basal: M 0.31 (0.14–0.50) P 0.16 (0.04–0.42) µmol/(100 ml forearm × min); clamp: M: 0.008 (−0.036 to 0.027) *P* −.025 (−0.076 to −0.001) µmol/(100 ml forearm × min), time (basal or clamp) × treatment *p* = .61, time *p* = .000, and treatment *p* = .36).

### 3.8 Lipolysis and systemic FFA

Systemic palmitate rate of appearance was not significantly different after M compared with P (M: 239 [204–275]; P: 278 [225–331], difference −39 [−97 to 19] µmol/min, *p* = .17). Also, there was no statistically significant interaction between time and treatment for FFA levels at *t* = 0, 100, 110, 120, 280, 290, and 300 (*p* = .99), and no main effect of treatment (*p* = .98), or genotype (*p* = .62).

### 3.9 Indirect calorimetry

RQ and glucose oxidation were increased and lipid oxidation were decreased during HEC compared with basal conditions, but there were no statistically significant differences in RQs, EE and rates of glucose oxidation between M and P (Table 3). Overall lipid oxidation was borderline significantly increased after M (main effect of treatment *p* = .08, 10% during basal [*p* = .10], and 9% during HEC [*p* = .34]). In addition, protein oxidation trended towards a decrease after M (main effect of treatment *p* = .082, 6% during the basal period [*p* = .44] and 24% during the HEC [*p* = .037]).

**Table 3:** Indirect calorimetry

	Placebo		Melatonin				
	Basal	HEC	Basal	HEC	Time × treatment	Treatment	Time
Respiratory quotient	0.77 [0.75–0.79]	0.84 [0.81–0.86]	0.77 [0.75–0.78]	0.83 [0.81–0.86]	0.96	0.72	0.00
Energy expenditure (kcal/24 h)	1769 (1726–1959)	1805 (1719–2019)	1820 (1730–2036)	1883 (1753–2027)	0.28	0.66	0.11
Lipid oxidation (kcal/24 h)	1207 [1077–1337]	808 [678–938]	1330 [1200–1460]	879 [749–1010]	0.62	0.080	0.00
Glucose oxidation (kcal/24 h)	240 [135–344]	614 [431–796]	197 [103–290]	617 [485–749]	0.61	0.73	0.00
Protein oxidation (kcal/24 h)	419 (268–575)	508 (297–555)	394 (245–520)	385 (167–519)	0.26	0.082	0.45

*Note*: Respiratory quotient, energy expenditure, lipid oxidation, glucose oxidation and protein oxidation derived from indirect calorimetry. *p* Values are provided for interactions between time (basal or HEC) and treatment (melatonin or placebo) and *p* values for main effect of treatment and time. Data are presented as medians (25%–75%) or means [95% confidence intervals] as appropriate.

Abbreviation: HEC, hyperinsulinemic‐euglycemic clamp.

### 3.10 Sleep quantity and quality

Total sleep time, sleep efficiency, and number of awakenings were not significantly different after M compared with P, but the actigraphy measured bedtimes were significantly delayed after M compared with P (*p* = .045) (Table 4). Of note, there were no indications of less total sleep time on the night before the study day in the melatonin group (−18 [−52 to 16] minutes after melatonin treatment compared with placebo [*p* = .28]). There were no significant effects of melatonin on daytime sleepiness or sleep quality measured with ESS (*p* = .84) or PSQI (*p* = .11), respectively, but a small shift towards eveningness type was seen after melatonin treatment compared with placebo measured with MEQ (difference −2.3 [−3.7 to −0.9], *p* = .0036) (Table 4).

**Table 4:** Sleep quantity and quality

	Placebo	Melatonin	Difference	*p*
Total sleep time (min)	421 [389–454]	412 [372–‐452]	−9 [−‐32 to 14]	0.40
Total time in bed (min)	478 [447–510]	463 [427–501]	−14 [−37 to 9]	0.20
Efficiency (%)	88 [86–90]	88 [85–92]	1 [−1.5 to 2.5]	0.53
Latency (min)	0.81 [0.2–1.4]	0.82 [0.4–1.3]	0.01 [−0.5 to 0.5]	0.96
Wake after sleep onset (min)	56.3 [47–65]	51 [38–63]	−5.1 [−14 to 3.7]	0.24
Number of awakenings	15.7 [14–18]	14.4 [12–17]	−1.3 [−3 to 1]	0.20
Average awakening time (min)	3.6 [3.1‐4.2]	3.6 ± [2.7–4.5]	−0.04 [−0.7 to 0.6]	0.88
In bed	22:52 [22:29–23:15]	23:23 [22:45–00:01]	32 [1–63] min	0.045
Out bed	6:50 [6:20–7:20]	7:07 [6:33–7:41]	17 [−19 to 53] min	0.31
MEQ	65 [61–69]	63 [58–67]	−2 [−4 to −1]	0.004
PSQI	5.5 [4.1–7.0]	4.9 [3.5–6.2]	−0.6 [−1.5 to 0.2]	0.11
ESS	7.4 [5.3–9.4]	7.5 [5.6–9.3]	0.1 [−1 to 1]	0.84

*Note*: Means and differences (95% confidence interval [CI]) for sleep quality and quantity variables derived from 1 week of actigraphy before each study day and for Morningness‐Eveningness questionnaire (MEQ), Pittsburgh Sleep Quality Index (PSQI), and Epworth Sleepiness Scale (ESS) questionnaires after 12 weeks of treatment. *p* values are from paired *t*‐tests between melatonin and placebo.

### 3.11 Routine biochemistry

There were no changes between M and P for any of the routine biochemical variables (Supporting Information: Table S2).

### 3.12 Other hormones

Leptin concentrations was increased after melatonin treatment compared with placebo (*p* = .041). Other hormones are available in Supporting Information: Table S3.

### 3.13 Western blot

Akt is a downstream target of the insulin receptor activated by phosphorylation. It is required to mediate the effects of insulin on glucose transporter 4 (GLUT4) translocation to the plasma membrane.^31^ There were no statistically significant interactions between treatment and time (basal or HEC conditions) or main effect of treatment (Supporting Information: Table S4) and there was no order‐of‐treatment effect (*p* = .54). Glycogen synthase is responsible for glycogen synthesis and is inactivated by phosphorylation.^32^ There were no statistically significant interactions between treatment and time or main effect of treatment and there was no order‐of‐treatment effect (*p* = .37). In addition, the ratio between the protein amount of GLUT4 and Hexokinase 2 to total membrane protein content did not change after treatment and there was no order‐of‐treatment effects (*p* = .65 and *p* = .50, respectively).

### 3.14 Harms

During melatonin treatment, one serious adverse event (SAE) (erysipelas) and six adverse events (AE) most likely unrelated to melatonin treatment occurred. During placebo treatment two SAE (colorectal carcinoma and severe psychiatric disease) and one AE occurred. The AEs have been listed in the supplementary information.

## 4 DISCUSSION

This is the first randomized, placebo‐controlled trial addressing the impact of long‐term, bedtime melatonin treatment on insulin sensitivity and β‐cell function in male patients with type 2 diabetes. In a group of patients with reduced insulin sensitivity, we report a further 12% reduction in insulin sensitivity after melatonin treatment compared with placebo measured by the HEC method. The significantly increased insulin levels during the SPIS of the IVGTT after melatonin treatment, despite similar levels of glucose, support reduced insulin sensitivity after melatonin treatment. Interestingly, the decrease in insulin sensitivity is accompanied by an increase in leptin levels, which previously have been shown to be inversely correlated with whole‐body glucose disposal.^33^ However, the decrease in insulin sensitivity was not accompanied by a reduction of skeletal muscle insulin signaling or total protein amount of GLUT4 or hexokinase 2; but of course, insulin resistance could have been induced through other pathways that we did not investigate. It is also possible that the negative effect of melatonin on insulin sensitivity is exerted by the elevated daytime melatonin levels observed after melatonin treatment. We have previously shown that pharmacological melatonin levels during daytime (approximately 10.000 pg/ml) reduces insulin sensitivity in healthy young males by 9%.^9^ However, as no dose‐response studies have been performed, the effect could also be evident at <32 pg/ml. Still, it was not the individuals with the highest melatonin levels that had the largest reduction in insulin sensitivity. In future studies, the elevated levels of melatonin during the study day can be avoided if the participants omit their intake of melatonin on the night before the study day.

One could argue that the dosage of 10 mg was too high when melatonin levels were still elevated during daytime. Yet, comparable daytime levels of melatonin have also been observed after treatment with only 2.5 mg of melatonin before bedtime.^34^ A dose of 10 mg is two to five times higher than usually prescribed dosages,^35^ but 10 mg tablets are available without prescription and are used to a considerable extend in larger markets such as the United States,^36^ why we found it relevant to investigate this dosage. Possibly, the dose of 10 mg could be associated with negative effects of melatonin treatment whereas lower doses could have a beneficial effect as suggested by the effects of melatonin on fasting plasma glucose, insulin, and HOMA‐IR detected in a recent meta‐analysis by Delpino et al. (dose range 3–10 mg).^37^ Yet, this cannot explain the discrepancy entirely as sensitivity analyses by Delpino et al. found that the positive effects of melatonin treatment were also evident at dosages of 10 mg. Given that fasting plasma glucose, insulin and HOMA‐IR mostly reflect the insulin sensitivity of the liver whereas the M‐value obtained from the HEC predominantly reflects the peripheral insulin sensitivity^38^ we propose, that melatonin might have different effects on hepatic and peripheral insulin sensitivity.

Our data do not support that melatonin has a more deleterious effect on glucose metabolism in rs10830963 risk allele carriers compared to wildtype allele carriers in any of our prespecified analyses. However, risk allele carriers had almost fourfold increased melatonin levels during daytime compared with wild‐type carriers. Additionally, there was no effect of the genotype in any of the pre‐specified analyses but a type 2 error, given the small sample size and the relatively small effect sizes observed in the previous studies,^4^, ^5^, ^6^, ^7^, ^8^ most likely explains this discrepancy. Furthermore, our genotype‐specific analyses are limited by the baseline difference in statin treatment and HbA1c where the risk‐allele carriers seemed to be more metabolically healthy than the wildtype carriers, which could perturb the interaction analyses.

Overall, our data suggest that melatonin increases fat oxidation and reduces protein oxidation. This is in accordance with the previously reported decrease in fat mass and increase in lean body mass after melatonin treatment (1 or 3 mg) for 1 year compared with placebo in healthy postmenopausal women.^39^ However, there was no significant effect of melatonin on body composition in our study. Contrary, a meta‐analysis by Delpino et al. found that melatonin significantly reduced total body weight.^40^ However, sensitivity analyses revealed that this only occurred in the low melatonin dose group (≤8 mg/day) which may explain the lack of effect in our study where 10 mg was used. As we observed no significant change in whole‐body lipolysis during the basal period as investigated with the palmitate tracer dilution technique, it is possible that the lipolysis fueling the increased fat oxidation is occurring either in the intramyocellular fat compartments or in the intramuscular adipocytes. The latter is supported by preclinical studies showing increased lipolysis and reduced lipid‐droplet size in intramuscular adipocytes after melatonin treatment.^41^

In our study, melatonin treatment did not increase total sleep time nor did it improve sleep efficiency compared with placebo in patients with type 2 diabetes. In accordance with our findings, a previous meta‐analysis in patients with insomnia found no effects of melatonin on total sleep time when measured with actigraphy or polysomnography.^42^ However, the point estimates for the MEQ were significantly reduced after melatonin compared with placebo, but the clinical importance of such a minor shift (two‐point reduction on a scale from 16 to 86) toward an evening chronotype type is debatable. Yet, it could reflect that we administered melatonin in a way that resulted in a slight delay in the circadian phase. This is supported by the actual bedtimes assessed by actigraphy, where the participants during melatonin treatment went to bed half an hour later than during placebo treatment. As evening chronotype is associated with diabetes and metabolic syndrome,^43^ this shift toward an evening chronotype could be one of the mechanisms underlying the decrease in insulin sensitivity observed in our study; it would therefore be of major interest to repeat the current study with an advanced timing of melatonin administration. Of note, the participants slept averagely 18 min less on the night before the study day during melatonin treatment and we cannot exclude that this reduction in total sleep time could drive some of the negative effects of melatonin on insulin sensitivity. Yet, the reduction in total sleep time was not statistically significant (*p* = .28) and could as well be attributed to random sampling variation.

Our current study is limited by the relatively small sample size that restricts conclusions drawn from the genotype‐specific analyses, but with the comprehensive study protocol employed, it is for practical reasons difficult to include a significantly higher number of participants. The crossover design used is a strength as well as a limitation. It eliminates the interindividual variability thus improving the power of the statistical analyses, but it also includes the risk of introducing carry‐over effects. As insulin sensitivity and β‐cell function are dynamic parameters as observed after exercise and sleep restriction,^45^, ^46^, ^47^ it is our opinion that 1 month of washout is sufficient to minimize a potential carry‐over effect. Taken together with no signs of order‐of‐treatment effects in the statistical analyses we deem it unlikely that the data derived from insulin sensitivity and β‐cell function measurements were perturbed by carry‐over effects, but we cannot exclude the possibility. In addition, as we only investigated male Caucasian patients with type 2 diabetes, we cannot extrapolate our results to other groups. The major strengths of the study are the exhaustive methods applied to assess β‐cell function and insulin sensitivity, the use of a placebo‐controlled, double‐blinded randomized design, and the use of muscle biopsies to investigate the direct effect of melatonin on insulin signaling and protein expression of key glucose‐metabolic enzymes in muscle tissue.

## 5 CONCLUSION

In our study, the treatment of male patients with type 2 diabetes with 10 mg of melatonin for 3 months before bedtime reduces insulin sensitivity. Given the worldwide increase in the use of synthetic melatonin, future studies are warranted to investigate whether these results can be translated to women with type 2 diabetes, healthy adults, and children and whether the effects of lower dosages of melatonin are similar. If our results are replicated, we recommend that patients with type 2 diabetes limit their use of melatonin in high doses, as reduced insulin sensitivity is central to the pathophysiology of type 2 diabetes.

## AUTHOR CONTRIBUTION

Esben S. Lauritzen, Niels Møller, Niels Jessen, Julie Støy, and Ulla Kampmann conceived the study. Esben S. Lauritzen, Lise‐Lotte Christensen, and Mette G. B. Pedersen researched data. E.S.L made the statistical analyses and drafted the manuscript. Esben S. Lauritzen, Niels Møller, Niels Jessen, Ulla Kampmann, Lise‐Lotte Christensen, Mette G. B. Pedersen,  and Julie Støy contributed to the discussion, reviewed and edited the manuscript.

## CONFLICT OF INTEREST

The authors declare no conflict of interest.

## Supporting information

Supporting information.

## Data Availability

The data that support the findings of this study are available on request from the corresponding author. The data are not publicly available due to privacy or ethical restrictions.

## References

[1] Brzezinski A . Melatonin in humans. N Engl J Med. 1997;336(3):186‐195. 8988899 10.1056/NEJM199701163360306

[2] Cajochen C , Krauchi K , Wirz‐Justice A . Role of melatonin in the regulation of human circadian rhythms and sleep. J Neuroendocrinol. 2003;15(4):432‐437. 12622846 10.1046/j.1365-2826.2003.00989.x

[3] Dawson D , Gibbon S , Singh P . The hypothermic effect of melatonin on core body temperature: is more better? J Pineal Res. 1996;20(4):192‐197. 8836952 10.1111/j.1600-079x.1996.tb00258.x

[4] Prokopenko I , Langenberg C , Florez JC , et al. Variants in MTNR1B influence fasting glucose levels. Nat Genet. 2009;41(1):77‐81. 19060907 10.1038/ng.290 PMC2682768

[5] Langenberg C , Pascoe L , Mari A , et al. Common genetic variation in the melatonin receptor 1B gene (MTNR1B) is associated with decreased early‐phase insulin response. Diabetologia. 2009;52(8):1537‐1542. 19455304 10.1007/s00125-009-1392-x PMC2709880

[6] Gaulton KJ , Ferreira T , Lee Y , et al. Genetic fine mapping and genomic annotation defines causal mechanisms at type 2 diabetes susceptibility loci. Nat Genet. 2015;47:1415‐1425. 26551672 10.1038/ng.3437 PMC4666734

[7] Mulder H , Nagorny CL , Lyssenko V , Groop L . Melatonin receptors in pancreatic islets: good morning to a novel type 2 diabetes gene. Diabetologia. 2009;52(7):1240‐1249. 19377888 10.1007/s00125-009-1359-y

[8] Lyssenko V , Nagorny CL , Erdos MR , et al. Common variant in MTNR1B associated with increased risk of type 2 diabetes and impaired early insulin secretion. Nat Genet. 2009;41(1):82‐88. 19060908 10.1038/ng.288 PMC3725650

[9] Kampmann U , Lauritzen ES , Grarup N , et al. Acute metabolic effects of melatonin‐a randomized crossover study in healthy young men. J Pineal Res. 2021;70(2):e12706. 33220095 10.1111/jpi.12706

[10] Cagnacci A , Arangino S , Renzi A , et al. Influence of melatonin administration on glucose tolerance and insulin sensitivity of postmenopausal women. Clin Endocrinol (Oxf). 2001;54(3):339‐346. 11298086 10.1046/j.1365-2265.2001.01232.x

[11] Rubio‐Sastre P , Scheer FA , Gomez‐Abellan P , Madrid JA , Garaulet M . Acute melatonin administration in humans impairs glucose tolerance in both the morning and evening. Sleep. 2014;37(10):1715‐1719. 25197811 10.5665/sleep.4088 PMC4173928

[12] Bonnefond A , Clément N , Fawcett K , et al. Rare MTNR1B variants impairing melatonin receptor 1B function contribute to type 2 diabetes. Nat Genet. 2012;44(3):297‐301. 22286214 10.1038/ng.1053 PMC3773908

[13] McMullan CJ , Schernhammer ES , Rimm EB , Hu FB , Forman JP . Melatonin secretion and the incidence of type 2 diabetes. JAMA. 2013;309(13):1388‐1396. 23549584 10.1001/jama.2013.2710 PMC3804914

[14] Clarke TC , Black LI , Stussman BJ , Barnes PM , Nahin RL . Trends in the use of complementary health approaches among adults: United States, 2002‐2012. Natl Health Stat Report. 2015;79:1‐16. PMC4573565 25671660

[15] Black LI , Clarke TC , Barnes PM , Stussman BJ , Nahin RL . Use of complementary health approaches among children aged 4‐17 years in the United States: National Health Interview Survey, 2007‐2012. Natl Health Stat Report. 2015;78:1‐19. PMC4562218 25671583

[16] Lauritzen ES , Kampmann U , Smedegaard SB , Støy J . Effects of daily administration of melatonin before bedtime on fasting insulin, glucose and insulin sensitivity in healthy adults and patients with metabolic diseases. A systematic review and meta‐analysis. Clin Endocrinol (Oxf). 2021;95:691‐701. 34370338 10.1111/cen.14576

[17] Abumrad NN , Rabin D , Diamond MP , Lacy WW . Use of a heated superficial hand vein as an alternative site for the measurement of amino acid concentrations and for the study of glucose and alanine kinetics in man. Metabolism. 1981;30(9):936‐940. 7022111 10.1016/0026-0495(81)90074-3

[18] Lauritzen ES , Jørgensen JOL , Møller N , Nielsen S , Vestergaard ET . Increased lipolysis after infusion of acylated ghrelin: a randomized, double‐blinded placebo‐controlled trial in hypopituitary patients. Clin Endocrinol (Oxf). 2020;93(6):672‐677. 32975853 10.1111/cen.14290

[19] DeFronzo RA , Tobin JD , Andres R . Glucose clamp technique: a method for quantifying insulin secretion and resistance. Am J Physiol. 1979;237(3):E214‐E223. 382871 10.1152/ajpendo.1979.237.3.E214

[20] Tripathy D , Wessman Y , Gullstrom M , Tuomi T , Groop L . Importance of obtaining independent measures of insulin secretion and insulin sensitivity during the same test: results with the botnia clamp. Diabetes Care. 2003;26(5):1395‐1401. 12716795 10.2337/diacare.26.5.1395

[21] Ferrannini E . The theoretical bases of indirect calorimetry: a review. Metabolism. 1988;37(3):287‐301. 3278194 10.1016/0026-0495(88)90110-2

[22] Whitney RJ . The measurement of volume changes in human limbs. J Physiol. 1953;121(1):1‐27. 13085295 10.1113/jphysiol.1953.sp004926 PMC1366051

[23] Mose M , Brodersen K , Rittig N , et al. Anabolic effects of oral leucine‐rich protein with and without β‐hydroxybutyrate on muscle protein metabolism in a novel clinical model of systemic inflammation‐a randomized crossover trial. Am J Clin Nutr. 2021;114(3):1159‐1172. 34081111 10.1093/ajcn/nqab148

[24] Garaulet M , Gómez‐Abellán P , Rubio‐Sastre P , Madrid JA , Saxena R , Scheer FAJL . Common type 2 diabetes risk variant in MTNR1B worsens the deleterious effect of melatonin on glucose tolerance in humans. Metabolism. 2015;64(12):1650‐1657. 26440713 10.1016/j.metabol.2015.08.003 PMC4856010

[25] Topp CW , Østergaard SD , Søndergaard S , Bech P . The WHO‐5 Well‐Being Index: a systematic review of the literature. Psychother Psychosom. 2015;84(3):167‐176. 25831962 10.1159/000376585

[26] Bech P , Rasmussen NA , Olsen LR , Noerholm V , Abildgaard W . The sensitivity and specificity of the Major Depression Inventory, using the present state examination as the index of diagnostic validity. J Affect Disord. 2001;66(2‐3):159‐164. 11578668 10.1016/s0165-0327(00)00309-8

[27] Johns MW . A new method for measuring daytime sleepiness: the epworth sleepiness scale. Sleep. 1991;14(6):540‐545. 1798888 10.1093/sleep/14.6.540

[28] Buysse DJ , Reynolds CF, 3rd , Monk TH , Berman SR , Kupfer DJ . The Pittsburgh Sleep Quality Index: a new instrument for psychiatric practice and research. Psychiatry Res. 1989;28(2):193‐213. 2748771 10.1016/0165-1781(89)90047-4

[29] Horne JA , Ostberg O . A self‐assessment questionnaire to determine morningness‐eveningness in human circadian rhythms. Int J Chronobiol. 1976;4(2):97‐110. 1027738

[30] Yeh KC , Kwan KC . A comparison of numerical integrating algorithms by trapezoidal, lagrange, and spline approximation. J Pharmacokinet Biopharm. 1978;6(1):79‐98. 650423 10.1007/BF01066064

[31] Mackenzie RW , Elliott BT . Akt/PKB activation and insulin signaling: a novel insulin signaling pathway in the treatment of type 2 diabetes. Diabetes Metab Syndr Obes. 2014;7:55‐64. 24611020 10.2147/DMSO.S48260 PMC3928478

[32] Nielsen JN , Richter EA . Regulation of glycogen synthase in skeletal muscle during exercise. Acta Physiol Scand. 2003;178(4):309‐319. 12864735 10.1046/j.1365-201X.2003.01165.x

[33] Haffner SM , Miettinen H , Mykkänen L , Karhapää P , Rainwater DL , Laakso M . Leptin concentrations and insulin sensitivity in normoglycemic men. Int J Obes Relat Metab Disor. 1997;21(5):393‐399. 10.1038/sj.ijo.0800419 9152742

[34] Scheer FA , Morris CJ , Garcia JI , et al. Repeated melatonin supplementation improves sleep in hypertensive patients treated with beta‐blockers: a randomized controlled trial. Sleep. 2012;35(10):1395‐1402. 23024438 10.5665/sleep.2122 PMC3443766

[35] Tordjman S , Chokron S , Delorme R , et al. Melatonin: pharmacology, functions and therapeutic benefits. Curr Neuropharmacol. 2017;15(3):434‐443. 28503116 10.2174/1570159X14666161228122115 PMC5405617

[36] Erland LA , Saxena PK . Melatonin natural health products and supplements: presence of serotonin and significant variability of melatonin content. J Clin Sleep Med. 2017;13(2):275‐281. 27855744 10.5664/jcsm.6462 PMC5263083

[37] Delpino FM , Figueiredo LM , Nunes BP . Effects of melatonin supplementation on diabetes: a systematic review and meta‐analysis of randomized clinical trials. Clin Nutr. 2021;40(7):4595‐4605. 34229264 10.1016/j.clnu.2021.06.007

[38] Tripathy D , Almgren P , Tuomi T , Groop L . Contribution of insulin‐stimulated glucose uptake and basal hepatic insulin sensitivity to surrogate measures of insulin sensitivity. Diabetes Care. 2004;27(9):2204‐2210. 15333485 10.2337/diacare.27.9.2204

[39] Amstrup AK , Sikjaer T , Pedersen SB , Heickendorff L , Mosekilde L , Rejnmark L . Reduced fat mass and increased lean mass in response to 1 year of melatonin treatment in postmenopausal women: a randomized placebo‐controlled trial. Clin Endocrinol (Oxf). 2016;84(3):342‐347. 26352863 10.1111/cen.12942

[40] Delpino FM , Figueiredo LM . Melatonin supplementation and anthropometric indicators of obesity: a systematic review and meta‐analysis. Nutrition (Burbank, Los Angeles County, Calif). 2021;91‐92:111399. 10.1016/j.nut.2021.111399 34626955

[41] Liu K , Yu W , Wei W , et al. Melatonin reduces intramuscular fat deposition by promoting lipolysis and increasing mitochondrial function. J Lipid Res. 2019;60(4):767‐782. 30552289 10.1194/jlr.M087619 PMC6446696

[42] Ferracioli‐Oda E , Qawasmi A , Bloch MH . Meta‐analysis: melatonin for the treatment of primary sleep disorders. PLoS One. 2013;8(5):e63773. 23691095 10.1371/journal.pone.0063773 PMC3656905

[43] Yu JH , Yun CH , Ahn JH , et al. Evening chronotype is associated with metabolic disorders and body composition in middle‐aged adults. J Clin Endocrinol Metab. 2015;100(4):1494‐1502. 25831477 10.1210/jc.2014-3754

[44] Borghouts LB , Keizer HA . Exercise and insulin sensitivity: a review. Int J Sports Med. 2000;21(1):1‐12. 10683091 10.1055/s-2000-8847

[45] Malin SK , Rynders CA , Weltman JY , Barrett EJ , Weltman A . Exercise intensity modulates Glucose‐Stimulated insulin secretion when adjusted for adipose, liver and skeletal muscle insulin resistance. PLoS One. 2016;11(4):e0154063. 27111219 10.1371/journal.pone.0154063 PMC4844153

[46] Donga E , van Dijk M , van Dijk JG , et al. A single night of partial sleep deprivation induces insulin resistance in multiple metabolic pathways in healthy subjects. J Clin Endocrinol Metab. 2010;95(6):2963‐2968. 20371664 10.1210/jc.2009-2430

[47] Mesarwi O , Polak J , Jun J , Polotsky VY . Sleep disorders and the development of insulin resistance and obesity. Endocrinol Metab Clin North Am. 2013;42(3):617‐634. 24011890 10.1016/j.ecl.2013.05.001 PMC3767932

