# Supplementary material for: Three months of melatonin treatment reduces insulin sensitivity in patients with type 2 diabetes—A randomized placebo‐controlled crossover trial
Source: J Pineal Res. 2022 Jun 9;73(1):e12809. doi: 10.1111/jpi.12809 (PMC9540532; doi:10.1111/jpi.12809)
Supplement: Supplementary file 1 — Supporting information. [file JPI-73-e12809-s001.docx]

**Supplementary Table S1 – incremental area under the curves for glucose, insulin, C-peptide and glucagon during the IVGTT**

|  | Placebo | Melatonin | Difference | P |
| --- | --- | --- | --- | --- |
| Glucose iAUC_120-130 min_  (mmol/l x min) | 101 [94-107] | 96 [90-103] | -5 [-11-2] | 0.17 |
|  |  |  |  |  |
| Glucose iAUC_130-180 min_  (mmol/l x min) | -117 (-128- -106) | -123 (-135- -112) | -6 [-18-5] | 0.27 |
|  |  |  |  |  |
| Insulin iAUC_120-130 min_  (pmol/l x min) | 153 (84-430) | 115 (20-467) | -21 (-124-30) | 0.15 |
|  |  |  |  |  |
| Insulin iAUC_130-180 min_  (pmol/l x min) | 1017 (-155-2311) | 1262 (771-3720) | 349 (55-1213) | 0.03 |
|  |  |  |  |  |
| C-peptide iAUC_120-130 min_  (pmol/l x min) | 737 (100-1452) | 374 (131-1589) | -180 [-510-150] | 0.26 |
|  |  |  |  |  |
| C-peptide iAUC_130-180 min_ (pmol/l x min) | 18646 (10785-25062) | 23437 (12414-26494) | 1695 (-1339-5500) | 0.10 |
|  |  |  |  |  |
| Glucagon iAUC_120-130 min_  (pmol/l x min) | -24 (-56 - -18) | -35 (-46- -18) | 3 (-15-12) | 0.89 |
|  |  |  |  |  |
| Glucagon iAUC_130-180 min_  (pmol/l x min) | -25 (-49- -11) | -5 (-35-5) | 13 [-9-35] | 0.24 |

**Table S2 legend:** *Incremental area under the curves (iAUC) for glucose, insulin, C-peptide and glucagon during the IVGTT stratified on the time during first-phase insulin secretion (iAUC time 120-130 min) and second-phase insulin secretion (iAUC time 130-180 min). Data are presented as medians (25-75 %) or means [95%-confidence intervals] as appropriate.*

**Supplementary Table S2: Clinical biochemistry, Weight, Blood pressure, and psychological health for the participants at week 0, week 6 and week 12.**

|  | Week 0 |  | Week 6 |  | Week 12 |  | time x treatment | treatment |
| --- | --- | --- | --- | --- | --- | --- | --- | --- |
| Clinical Biochemistry | Placebo | Melatonin | Placebo | Melatonin | Placebo | Melatonin |  |  |
| Total Cholesterol (mM) | 4.0 (3.5-4.6) | 3.9 (3.6-4.5) | 4.1 (3.6-3.6) | 4.0 (3.5-4.8) | 3.6 (3.4-4.2) | 3.6 (3.4-4.5) | 0.68 | 0.35 |
| HDL (mM) | 1.1 (1.0-1.4) | 1.1 (0-9-1.4) | 1.2 (1.0-1.4) | 1.1 (1.0-1.3) | 0.9 (0.9-1.3) | 1 (0.8-1.3) | 0.72 | 0.25 |
| LDL (mM) | 2.2 [1.8-2.5] | 2.1 [1.7-2.4] | 2.0 [1.7-2.4] | 2.1 [1.7-2.5] | 2.1 [1.8-2.4] | 2.1 [1.7-2.4] | 0.27 | 0.76 |
| Triglycerides (mM) | 1.6 (1.2-2.0) | 1.6 (0.9-2.5) | 2.0 (0.9-3.0) | 1.8 (1.0-2.9) | 1.4 (0.7-1.7) | 1.6 (0.9-2.1) | 0.57 | 0.07 |
| HbA1c (mmol/mol) | 48 [45-52] | 48 [45-51] | 49 [44-43] | 48 [44-51] | 50 [45-54] | 50 [46-54] | 0.74 | 0.70 |
| Hemoglobin (mM) | 9.3 [8.8-9.7] | 9.3 [8.6-9.9] | 9.2 [8.8-9.7] | 9.3 [8.9-9.7] | 8.7 [8.3-9.2] | 8.8 [8.4-9.2] | 0.77 | 0.74 |
| Anthropometrics | |  |  |  |  |  |  |  |
| Weight (kg) | 93 (86-102) | 94 (84-102) | 92 (87-103) | 95 (88-102) | 92 (84-103) | 93 (86-103) | 0.3 | 0.73 |
| Systolic blood pressure (mmHg) | 141 [135-148] | 137 [129-144] | 137 [132-143] | 135 [130-141] | 141 [135-147] | 136 [132-141] | 0.69 | 0.11 |
| Diastolic blood pressure (mmHg) | 86 [79-92] | 84 [79-89] | 86 [81-91] | 83 [78-88] | 83 [78-88] | 85 [79-90] | 0.23 | 0.46 |
| Psychological health | | |  |  |  |  |  |  |
| WHO-5 | 80 [73-86] | 82 [78-86] | 81 [73-89] | 82 [76-87] | 80 [73-87] | 80 [73-87] | 0.84 | 0.64 |
| MDI | 5 (1-7) | 4 (1-6) | 2 (1-7) | 2 (1-6) | 2 (2-7) | 3 (1-8) | 0.86 | 0.63 |

**Table S4 legend:** *Means [95 % CI] or median (25-75%) for clinical biochemical variables, anthropometric variables and for Major Depression Inventory (MDI) and World Health Organization Five Well-being Index (WHO-5). HDL = high-density lipoprotein, LDL = low-density lipoprotein.*

**Table S3 – other hormones**

|  | Placebo | Melatonin | Difference | P-value |
| --- | --- | --- | --- | --- |
| Leptin (µg/l) | 6.2 (4.8-12.3) | 7.2 (4.7-15.7) | 0.8 (-1-2.9) | 0.041 |
|  |  |  |  |  |
| Cortisol (ng/ml) | 122 [106-139] | 117 [102-132] | -5 [-24-13] | 0.56 |
|  |  |  |  |  |
| Ghrelin (pg/ml) | 35 (28-52) | 31 (28-55) | -3 [-13-7] | 0.56 |
|  |  |  |  |  |
| Adiponectin (mg/l) | 6.9 [5.3-9.1] | 6.8 [1.7-8.8] | 0.97 [0.9-1.1]* | 0.51 |
|  |  |  |  |  |
| GH (ng/ml) | 0.4 [0.2-0.9] | 0.4 [0.2-0.7] | 0.99 [0.6-1.7]* | 0.89 |
|  |  |  |  |  |
| IGF-1 (ng/ml) | 113 [98-129] | 124 [104-143] | 10 [-3-23] | 0.13 |
|  |  |  |  |  |
| IGFBP3 (µg/ml) | 2.3 [2.1-2.6] | 2.4 [2.1-2.7] | 0.1 [-0.1-0.2] | 0.57 |
|  |  |  |  |  |
| IGFBP1 (µg/l) | 18 [12-27] | 17 [11-27] | 1 [0.8-1.1]* | 0.57 |
|  |  |  |  |  |
| MBL (ng/ml) | 1109 (698-2331) | 1175 (615-2373) | 1 [0.9-1.1]* | 0.84 |
|  |  |  |  |  |
| hs-CRP (mg/l) | 1.2 (0.4-1.8) | 1.0 (0.5-2.6) | 0.5 [-0.1-1.5] | 0.11 |
|  |  |  |  |  |
| GLP-1 (pmol/l) | 7.4 [4.6-11.7] | 8.9 [5.2-15.2] | 1.2 [0.9-1.5]* | 0.11 |
|  |  |  |  |  |
| GIP (pmol/l) | 9.3 (6.3-15.9) | 8.4 (6.9-13.7) | -0.5 (-2-1.6) | 0.71 |

**Table S4 legend:** *Means [95 % CI] or medians (25-75%) for leptin, cortisol, acylated ghrelin, adiponectin, growth hormone (GH), Insulin-like growth factor 1 (IGF-1), Insulin-like growth factor binding protein 3 (IGFBP3), Insulin-like growth factor binding protein 1 (IGFBP1), Mannan-binding lectin (MBL) and high-sensitivity C-reactive protein (hs-CRP). *Back transformed ratio between melatonin and placebo.*

**Supplementary Table S4 – Western blot targets**

| Western Blot Target Ratios | Placebo |  | Melatonin |  | Treatment x time | Treatment | Time |
| --- | --- | --- | --- | --- | --- | --- | --- |
|  | Basal | Clamp | Basal | Clamp |  |  |  |
| GLUT4/total protein | 3.42 [2.8-4.1] | 3.44 [2.8-4.1] | 3.36 [2.7-4.0] | 3.49 [2.8-4.1] | 0.66 | 1 | 0.6 |
| GLUT4 | 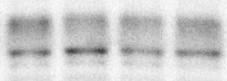 | | | |  |  |  |
| Hexokinase 2/total protein | 0.044 [0.024-0.063] | 0.046 [0.025-0.066] | 0.054 [0.030-0.079] | 0.036 [0.020-0.053] | 0.1 | 1 | 0.18 |
| Hexokinase 2 | 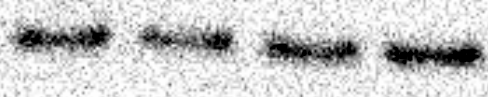 | | | |  |  |  |
| pAkt/total Akt | 0.45 [0.35-0.56] | 1.87 [1.44-2.30] | 0.45 [0.34-0.56] | 1.63 [1.23-2.03] | 0.56 | 0.52 | <0.000 |
| pAKT  total Akt | 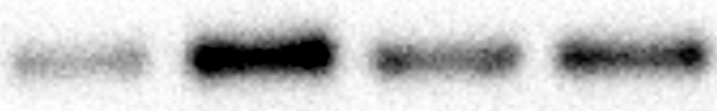  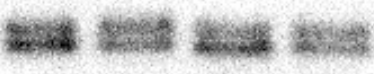 | | | |  |  |  |
| pGS/total GS* | 0.082 [0.065-0.098] | 0.044 [0.028-0.061] | 0.104 [0.086-0.121] | 0.057 [0.039-0.074] | 0.13 | 0.16 | <0.000 |
| pGS  Total GS | 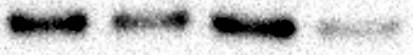  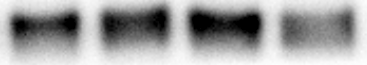 | | | |  |  |  |

**Table S6 legend:** *Western blot targets (Glucose Transporter Type 4 (GLUT4), Hexokinase 2, 473-Phospho-Serine Protein Kinase B (pAkt), total Protein Kinase B (Akt), 641-Phospho-Serine Glycogen Synthase (pGS), total Glycogen Synthase (GS) expressed as ratios between target and total membrane stain-free protein or phosphorylated target/total target. The western blot examples are from a participant who received the treatment in placebo-melatonin order. Data were analyzed with the linear mixed model with the fixed factor time defined as either HEC or basal period.*

**Supplementary Figure S1: Outline of the study design**


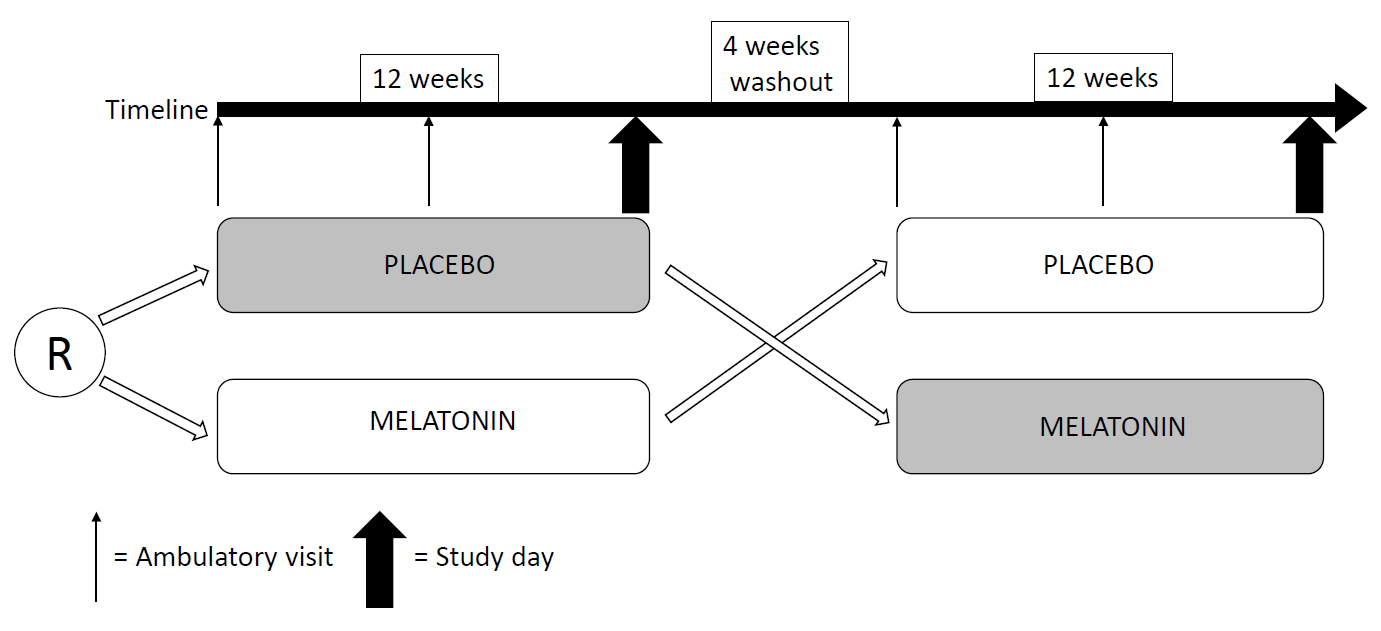


**Figure S1 legend:** Outline of the study design, R = randomization. MELATONIN = 10 mg melatonin 1 h before bedtime each night. PLACEBO = identical tablet with 10 mg glucose monohydrate 1 h before bedtime each night.

**Supplementary Figure S2: flowchart of participants**


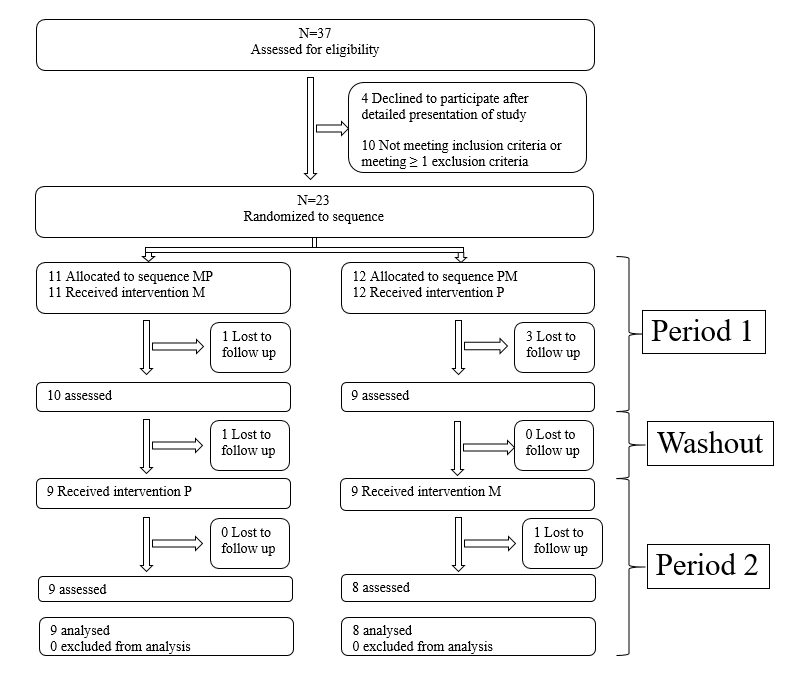


**Figure S2 legend:** *Flowchart of participants in the study. In the melatonin placebo (MP) sequence two was lost to follow up: one due to worsening in metformin-induced abdominal pain and diarrhea (M) and one was misdiagnosed with type 2 diabetes (the participant was GAD65 positive) (P). In the placebo melatonin (PM) sequence, four was lost to follow up: one developed thrombocytopenia and elevated liver enzymes (P), one got diagnosed with colon-rectal carcinoma (P), one developed severe psychiatric disease (P) and the last wanted to leave the project due to personal reasons (M).*

**Primary antibodies for the western blot analyses:** Hexokinase 2, #ab209847 (waiting for **RRID** registration), Abcam, Cambridge, United Kingdom. Glucose Transporter 4 (GLUT4), #07-1404, **RRID:AB_1587080,** Merckmillipore, Darmstandt, Germany. pAkt (ser473), #9271, **RRID:AB_329825**; pGS (ser641) #3891, **RRID:AB_2116390**; total GS #3886, **RRID:AB_2116392,** Cell Signaling Technology, Danvers, MA, USA. Total Akt, #MA5-14918, **RRID:AB_10989513,** Thermofisher, Waltham, MA, USA.

**Adverse events:** Melatonin treatment (6): 1: drop in haemoglobin associated with removal of benign colon polyp; 1: spot serum cortisol > 550 nM: normal CT of adrenal glands and overnight dexamethasone suppression test; 1: abdominal pain and diarrhoea, resolved after cessation of metformin therapy (excluded, see **Figure S2**); 1: diagnosed with LADA (HbA1c increased steadily from 40 to 68 mmol/mol without any changes in treatment, positive GAD65 antibodies, excluded, see **Figure S2**); 2: diarrhoea resolved after cessation of melatonin). Placebo treatment (1): (1 thrombocytopenia and elevated liver enzymes – resolved spontaneously, excluded, see **Figure S2**))
